# Effects of twin-block appliance on quality of life in OSA children with class 2 malocclusion and mandibular retrognathia

**DOI:** 10.1016/j.jobcr.2025.06.003

**Published:** 2025-06-13

**Authors:** Maen Zreaqat, Sahal Alforaidi, Rozita Hassan

**Affiliations:** a Orthodontic Dep. School of Dental Sciences, Universiti Sains Malaysia, Malaysia; b Pediatric Dentistry and and Orthodontics Dep. College of Dentistry, Taiba University, Saudi Arabia

**Keywords:** Quality of life, Sleep apnea, Twin block

## Abstract

**Purpose:**

Twin block has been proposed as a potential oral appliance for the treatment of obstructive sleep apnea (OSA) in children with class 2 skeletal malocclusion. This study aimed to evaluate the impact of twin block appliance on quality of life in children with OSA.

**Materials and methods:**

This case series study comprised 47 growing OSA children with Class 2 skeletal malocclusion and mandibular retrognathia aged 10–12 years. Patients were treated with a functional twin block appliance and the quality of life was assessed using the OSA-18 questionnaire, which was administered before appliance insertion and 30 days after the end of treatment. Additionally, a second standard overnight polysomnography (PSG) was performed to determine changes in respiratory parameters.

**Results:**

All domains of the OSA-18 questionnaire showed significant changes (P < 0.025), except for physical suffering domain (P = 0.075). The mean total scores of the OSA-18 questionnaire decreased from 81.55 to and 53.07 scores (28.49 scores, P < 0.001). At the end of the treatment, the apnea-hypopnea index decreased significantly by 4.53 events/h (74.0 %), P < 0.001).

**Conclusion:**

The twin block appliance resulted in a significant improvement in four of the five domains of the OSA-18 questionnaire, with a positive impact on the quality of life in OSA children with Class 2 skeletal malocclusion. Patients showed significant improvements in respiratory sleep parameters and parent-reported symptoms.

## 1 Introduction

Obstructive sleep apnea (OSA) is a prevalent sleep disorder characterized by partial or complete obstruction of the upper airway during sleep, resulting in disrupted breathing patterns and frequent awakenings.^1^ Although OSA affects individuals of all age groups, its occurrence in children is particularly concerning due to its potential long-term consequences on their growth, development, and overall well-being.^2^^,^^3^ OSA negatively affects the emotion, cognitive function, and behavior of children and their family members, which may contribute to a poor quality of life.^4^^,^^5^

Interceptive orthodontic treatment has been suggested as a potential management option for children.^6^ Rapid maxillary expansion (RME), mandibular advancement devices (MAD), and functional appliances (FA) have been proposed as management options for mild and moderate pediatric OSA. Evidence has shown that these approaches can diminish some OSA symptoms and improve respiratory parameters. A systematic review had reported a reduction in the apnea-hypopnea index (AHI) of approximately 6 points (from 11 to 5) after treatment with RME, FA, and MAD in mild and moderate cases.^7^ Minimum oxygen saturation increased following RME and FA.^8^ The twin block appliance is an orthodontic functional oral appliance designed to correct the dento-skeletal discrepancy in children with class 2 malocclusion and mandibular retrognathia by advancing the mandible and/or tongue with variable degrees of downward mandibular rotation.^9^^,^^10^ It is the most commonly applied functional orthodontic appliance owing to its high acceptance rate by patients and the ease of speech and mastication functions with its use, as the upper and lower units are separate.^11^

High-quality evidence affirming the effectiveness of orthodontic oral appliances in the treatment of pediatric OSA is limited and obscured by the methodological limitations of the available literature. However, the forward posture of the mandible and hyoid bone following treatment with twin block has been suggested to improve upper airway patency and respiratory parameters.^12^^,^^13^ Some reports have demonstrated significant reductions in the AHI, improved oxygen saturation levels, and enhanced airflow.^14^^,^^30^ However, evidence regarding the effect of twin block therapy on quality of life in children with OSA is still lacking. Therefore, this study aimed to explore the effect of twin block appliance treatment on quality of life in children with OSA using the OSA-18 questionnaire.

## 2 Materials and Methods

This clinical prospective cohort study was approved by the Research and Ethics Committee of the University (USM/JEPeM/20060315-November 2020). Informed consent was obtained from the participants’ parents before the assessment, and explanatory letters explaining the study were provided. Additionally, a simplified version of informed consent form for children, suitable for their readability standards, was developed. This study included a consecutive sample of growing children recruited from a private orthodontic clinic after being referred by a sleep physician. Data collection was performed over 30 months (June 2021 to November 2023). The inclusion criteria were the following:

• Mixed dentition in the age range of 10–12 years before their peak pubertal growth spurt, with stages 2 and 3 of cervical vertebral maturation.^15^
• Class 2 skeletal malocclusion (4.5° ≤ ANB≤ 8.0°) associated with normal maxilla (SNA, 79–84°) and mandibular retrusion (SNB ≤76°). The aforementioned ANB angle range matched the norms of the current sample (Saudi children). In fact, floating norms of ANB angle vary among different populations based on their craniofacial phenotypes.^16^
• Patients for whom functional twin block therapy had been indicated.
• Frankfort mandibular plane angle (FMA) in the range of 20–28° and overjet of 6–10 mm, with minimal crowding or spacing in either arch.
• Polysomnography (PSG)-proven OSA (AHI >1.0/h); PSG was performed as an
  overnight testing in a sleep laboratory with a trained health care professional in attendance.

The exclusion criteria of the study were as follows:

• History of adenotonsillectomy or orthodontic treatment
• The presence of an open bite
• Contraindications to functional treatment

Assuming that a mean change of 5 points in the total OSA-18 score would indicate a significant difference between pre- and post-treatment, the required sample size was calculated to be 47 subjects in order to achieve 80 % study power with a 5 % type I error rate. The standard deviation (2.37) used in this calculation was obtained from a previous study that evaluated the effects of rapid maxillary expansion (RME) on the quality of life in children with persistent snoring following adenotonsillectomy.^17^ The following baseline variables were assessed: overjet, cephalometric measurements, tonsil size (evaluated in the sitting position using the Friedman grading scale), and Mallampati score which comprises a visual assessment of the distance from the tongue base to the roof of the mouth and may serve as a clinical indicator of the presence and severity of OSA.^18^ Lateral cephalometric radiographs were obtained with the patients seated in an upright position, the Frankfort horizontal plane parallel to the floor, and the teeth in centric occlusion. Cephalograms were digitized with a MICROTEK ScanMaker4 scanner, while landmarks identification and measurements were analyzed using Dolphin Imaging and Management Solution Software, version 11.0 (Chatsworth, CA).

Individually customized twin block appliances were fabricated for each patient. One-step mandibular advancement was performed during the wax check bite, which was recorded with an edge-to-edge incisor relationship and a 3-mm opening between the maxillary and mandibular incisors. Single-phase advancement was proposed to maximize the orthopedic effect of the twin block appliance, despite the reduced patient compliance and comfort compared with the incremental advancement method.^19^ The bite blocks were trimmed to ensure normal vertical development of the mandibular buccal segments. Patients were instructed to wear the appliance full-time, even during mealtimes, whenever possible. To ensure treatment adherence, parents were asked to complete a daily wear-time assessment booklet. All patients were assessed monthly for 9 months. An Arabic validated version of the OSA-18 questionnaire ^20^ was administered before appliance insertion and 30 days after the end of the treatment to minimize the effects of muscle tension. Questionnaires were filled in by parents and instructions for completing the questionnaire were provided by the same operator. The questionnaire comprises 18 items grouped into five domains, where each item is scored on an ordinal 7-point classification (1 = none of the time, 2 = hardly any of the time, 3 = a little of the time, 4 = some of the time, 5 = a good bit of the time, 6 = most of the time, and 7 = all of the time). The OSA-18 domains are as follows: a) sleep disturbances (four items), b) physical suffering (four items), c) emotional distress (three items), d) daytime problems (three items), and e) parent or caretaker concern (four items). Based on this ordinal 7-point classification, the total OSA-18 score ranged from 18 to 126, and the impact on the QOL of children was categorized into three groups: minor (scores <60), moderate (scores between 60 and 80), and major (scores >80). The OSA-18 has been used as a screening tool and treatment outcome parameter for pediatric OSA in several studies.^21^^,^^22^

Additionally, a second overnight PSG testing was performed by the same sleep professional for the study group to assess the effects of twin block therapy on sleep parameters. PSG values were manually scored using the American Academy of Sleep Medicine 2012 Pediatric Criteria. Cephalometric data, tonsil size, Mallampati score, and body mass index (BMI) were reassessed.

## 3 Statistical analysis

Data were analyzed using the Statistical Package for the Social Sciences (SPSS) software (version 20.0, SPSSInc., Chicago, Ill, USA). The paired measurements were tested using the Shapiro–Wilk test and were normally distributed. A paired *t*-test was used to determine the changes in the OSA-18 scores after treatment. Statistical significance was set at P < 0.05.

## 4 Results

Fifty patients, with a mean age of 11.16 ± 1.39 years, were enrolled in this study (29 boys and 18 girls). The mean daily wear time of the twin block appliance was 13.48 ± 3.79 h/24 h. Three questionnaires were excluded because patients did not wear the appliance or due to false/incomplete survey scoring. Table 1 shows the mean cephalometric data, PSG variables, BMI, overjet, tonsil size, Mallampati score, corresponding standard deviations, and differences after treatment. Results showed that treatment with twin block appliance caused mandibular protrusion as SNB increased by 1.65° (P = 0.017), ANB (anteroposterior relationship of the mandible to the maxilla) decreased by 1.99° (P < 0.001), and mandibular length increased by 3.19 mm (P < 0.001). In addition, The overjet decreased by 4.87 mm (P < 0.001). However, changes in SNA° and maxillary arch length were not significant (P > 0.05). Both tonsil size and Mallampati score decreased significantly (P = 0.034 and 0.009 respectively). Moreover, most of PSG variables improved significantly after treatment; sleep efficiency increased by 3.44 % (P = 0.025), the oxygen desaturation index decreased by 5.36 events/h (P = 0.032), minimum oxygen saturation increased by 5.32 % (P < 0.001), arousal index decreased by 7.46 events/h (P < 0.001). The AHI decreased by 4.53 events/h (74 %, P < 0.001); in six children, the AHI returned to normal values (AHI<1) while 38 children had residual AHI (AHI>1), two children demonstrated a worsening in their AHI, and one patient had no response to treatment.

**Table 1:** Changes skeletal, dental, and respiratory variables among the study group.

Variable	OSA Pre-treatment Mean ± SD n = 47 Age = 11.16 ± 1.39	Post-treatment Mean ± SD	P Value
Sex			
Male	29	27	
Female	18	17	
BMI (kg/m2)	26.67 ± 3.74	25.93 ± 3.61	0.320
Overjet (mm)	7.70 ± 2.04	2.83 ± 1.17	<0.001
Tonsil size	2.29 ± 0.92	1.43 ± 0.85	0.024
Mallampati score	1.90 ± 0.87	1.13 ± 0.49	0.009
**Cephalometric data**			
SNA (°)	80.71 ± 4.07	80.37 ± 3.94	0.856
SNB (°)	75.24 ± 3.67	76.89 ± 3.84	0.017
ANB (°)	5.47 ± 1.88	3.48 ± 1.13	<0.001
FMA (°)	24.38 ± 2.32	25.43 ± 2.68	0.097
Maxillary length (ANS-PNS) (mm)	51.14 ± 3.60	51.95 ± 3.71	0.437
Effective mandibular length (Co-Gn) (mm)	94.35 ± 6.68	97.54 ± 6.84	<0.001
**PSG Variables**			
Total sleep time (h)	7.91 ± 1.40	7.94 ± 1.39	0.362
Sleep efficiency (%)	88.13 ± 0.70	91.57 ± 0.83	0.025
REM sleep %	18.30 ± 4.74	18.64 ± 4.39	0.462
Apnea Hypopnea index (events/h)	6.12 ± 3.65	1.59 ± 1.22	<0.001
Oxygen desaturation index (events/h)	12.68 ± 8.24	7.32 ± 2.30	0.032
Lowest SpO2 (%)	89.38 ± 4.36	94.70 ± 2.14	<0.001
Average SpO2 (%)	94.54 ± 2.22	94.89 ± 2.19	0.132
Arousal index (events/h)	16.81 ± 5.91	9.35 ± 2.40	<0.001

P < 0.05.

Table 2 shows the total and domain-specific pre- and post/treatment scores of the OSA-18 questionnaire. The domain of sleep disturbances decreased by 8.85 scores (P < 0.001), emotional distress decreased by 5.71 scores (P < 0.001), daytime problems decreased by 2.74 scores (P = 0.007), and caretaker concern decreased by 7.59 scores (P < 0.001). However, the change in the domain of physical suffering was not significant (3.60 scores, P = 0.075). The mean total scores of the OSA-18 questionnaire decreased from 81.55 to and 53.07 scores (28.49 scores, P < 0.001).

**Table 2:** Total and domain pre and postoperative scores - OSA-18 Questionnaire.

OSA-18 domain	Pre-operative OSA-18 survey	post-operative OSA-18 survey	Mean difference	P value
**Sleep disturbances**	20.88	12.03	−13.85 ± 3.54	<0.001
loud snoring?	5.87	3.82	−4.05 ± 1.92	0.014
periods in which breathing stopped or air seemed trapped during the night?	4.55	2.63	−2.92 ± 0.87	<0.001
choking noise or breathlessness while sleeping?	5.36	2.55	−3.81 ± 1.40	<0.001
restless sleep or frequent awakening during sleep?	5.10	3.03	−3.07 ± 1.26	<0.001
**Physical suffering**	17.17	13.57	−9.76 ± 2.28	0.075
mouth breathing due to nasal obstruction?	5.14	3.42	−2.99 ± 1.04	0.033
frequent common colds or upper airway infection?	4.07	3.67	−2.40 ± 0.91	0.079
nasal secretion or runny nose?	4.27	3.51	−2.39 ± 1.17	0.114
eating difficulties?	3.69	2.97	−1.98 ± 0.74	0.092
**Emotional distress**	13.10	7.39	−5.71 ± 1.73	<0.001
change in humor or rage?	4.57	2.47	−2.10 ± 1.03	<0.001
aggressive or hyperactive behavior?	4.10	2.68	−1.42 ± 0.61	<0.001
problems with discipline?	3.43	2.24	−1.19 ± 0.54	0.043
**Daytime problems**	9.32	6.58	−3.34 ± 1.45	0.007
drowsiness or excessive daytimenaps?	2.07	1.45	−0.62 ± 0.11	0.026
poor concentration or attention?	3.74	2.17	−1.57 ± 0.43	0.014
difficulty to wake up in the morning?	3.51	2.96	−1.15 ± 0.38	0.086
**Caretaker concern**	21.09	13.50	−11.70 ± 2.87	<0.001
leave you worried about the general health of your child?	5.36	3.49	−2.96 ± 1.20	0.012
created a concern that your child is not breathing enough air?	4.85	2.83	−3.02 ± 1.33	<0.001
interfered in your ability to carryout your daily activities?	4.92	3.43	−2.49 ± 1.06	0.009
made you feel frustrated?	5.96	3.75	−3.21 ± 1.41	0.004
Total OSA	81.55	53.07	−44.36 ± 9.52	<0.001

P < 0.05.

## 5 Discussion

Several studies have demonstrated the effects of OSA therapy on quality of life in children with this condition. However, most of these reports focused on the effect of adenotonsillectomy.^23^ The literature shows that the impact of twin block appliance on quality of life in children with OSA has not been assessed in any pediatric population; to the best of our knowledge, this is the first study to investigate this impact on growing children using the OSA-18 questionnaire.

The significant increase in SNB angle, significant decrease in ANB angle, and overjet reduction in the study group demonstrates the clinical effectiveness of the twin block appliance in the anteroposterior skeletal correction of class 2 malocclusion. However, the orthopedic effect exerted by the appliance on the maxilla was minimal (insignificant decrease in SNA° and increase in maxillary arch length) which is in concordance with other reports.^24^^,^^25^ Other studies, found a reciprocal restraining effect on the maxillary growth with the twin block appliance (headgear effect) due to the stretch of the facial muscles.^26^

The significant decrease in tonsil size and Mallampati score may show the favorable effect of twin block appliance in improving upper airway patency and respiratory parameters. However, part of these therapeutic effects may be attributed to the growth of the upper airway or regression of lymphoid tissues. Tonsils are present at birth and enlarge throughout childhood, reaching peak size by age 7 and then gradually shrink or regress as individuals approach adulthood.^27^

Sleep efficiency, which is the amount of time individuals spend sleeping while in bed, increased by 3.44 % (P = 0.025) after twin block therapy, indicating improved sleep quality. Additionally, AHI, the most important PSG parameter, decreased by 4.53 events/h (74 % decrease, P < 0.001), although only six patients had their AHI return to normal pediatric reference values (AHI<1, P = 0.214). This reduction may indicate the efficacy of twin block appliances in improving upper airway patency and respiratory parameters. A threshold of ≥50 % in reduction of the AHI was considered the criterion for response to treatment.^28^ Reduction in the AHI after OSA treatment with oral appliances have been reported in previous studies on sleep. However, the reported degree of AHI change after treatment varies considerably. Zhang et al. reported an average AHI decrease of 75.9 % after twin block appliance therapy for pediatric OSA,^29^ whereas others reported lower values of AHI reduction: 63.4 % by Villa et al.^30^ and 28.6 % by Umemoto et al.^31^ Patient compliance might partially explain these conflicting findings, as patients wearing the twin block appliance might have a more stable and favorable muscle function against upper airway collapsibility compared with non-compliant patients. In addition, the structure of the twin block appliance may not provide a firm protrusive position for the mandible and an enlarged pharyngeal airway compared to that achieved with a one-piece fixed appliance. The favorable changes in tonsil size and Mallampati scores after treatment may be explained by improved airflow, which may reduce the collapsibility of the pharyngeal airway and alleviate irritation of the airway-associated lymphoid tissues.

The current study showed that twin block appliance therapy resulted in significant improvement in four of the five domains of the OSA-18 questionnaire, including sleep disturbances, emotional distress, daytime problems, and caretaker concerns. The improvement in the physical suffering domain did not reach statistical significance (P = 0.075), which may reflect more frequent upper respiratory tract infections due to nasal obstruction or enlarged lymphoid tissue in children with OSA. The change in total scores of the OSA-18 questionnaire was 28.49, reflecting a mild-to-moderate impact on quality of life. The positive impact of twin block on quality of life in this study might be attributed to satisfaction with the treatment consequences among children and their families. An advancing mandible caused by the twin block reduces overjet and may improve a patient's facial profile. Additionally, improvement in respiratory parameters recorded after the end of treatment may indicate alleviation of sleep symptoms, which may positively affect the survey score despite the discomfort and extensive range of mild side effects at the beginning of treatment. Cozza et al. proposed a modified monobloc orthodontic appliance that positioned the mandible anteriorly into an edge-to-edge incisor relationship in 4–8-year-old children with OSA. Their results showed improvements in subjective sleep quality and daytime performance in children.^32^ In pediatric OSA, several trials have evaluated the impact of surgery (adenotonsillectomy) on quality of life. These studies showed that surgery offers patients important cognitive and intellectual benefits despite the recurrence of sleep symptoms after surgical intervention in some cases.^33^ Caixeta et al., in 2021, found that adenotonsillectomy had a positive impact on quality of life, where the improvement remained for at least 22 months after surgery.^34^

In adults, quality of life has been assessed after continuous positive airway pressure (CPAP) and MAD therapy. Both modalities have a significant influence on quality of life and have been proven to enhance functional and behavioral outcomes in adults with mild-to-moderate OSA. Although MAD therapy is less effective than CPAP in treatment outcomes, it has a higher reported compliance and adherence to therapy than that observed in CPAP, with a better tolerance.^35^ In 2004, Barnes et al. found that treatment with mandibular advancement splint MAD improved quality of life as measured by the Functional Outcomes of Sleep Questionnaire mean score and social outcome domain and by the 36-Item Short Form Survey (SF-36).^36^ Gagnadoux et al., in 2009, evaluated the effect of mandibular advancement devices on quality of life using Nottingham Health Profile and found significant improvement in four out of six domains including physical mobility, pain, emotional reaction, and sleep.^37^ In contrast, Marklund et al., in 2015, found no significant effects of oral appliance therapy on quality of life; however, their results may be biased by the short term of the course treatment (4 months only).^38^ In fact, most MADs are used in the treatment of adult OSA; however, their application in growing children or adolescents is limited due to the dramatic skeletal and dentoalveolar changes and overbite alterations they result in, which preclude their widespread use. Instead, functional orthodontic appliances are used where occlusal changes are favorable, particularly in children with class 2 malocclusion and mandibular retrognathia.

Interestingly, the current study showed a very good daily wear time (13.48 h/24 h), which was above the 8-h threshold required to achieve good patient compliance.^39^ However, patients’ compliance and adherence to the treatment protocol seem to be lower in younger children, and largely related to parental cooperation.^40^

The major limitation of the current study was the lack of a control group; natural growth and development in this age group may have affected airway dimensions. Notably, some changes in the AHI can be attributed to the growth of the upper airway or regression of lymphoid tissues. Therefore, changes that would have occurred in the absence of any treatment are uncertain. Moreover, the same operator provided the appliance and the survey which may raise concerns about the potential of self-reporting bias; however, the OSA-18 questionnaire is a validated self-reporting with a minimized recall bias due to the short exposure intervals. Overall, the twin block appliance may be advantageous in improving quality of life and respiratory parameters, although consistent agreement is elusive.

## 6 Conclusions

The twin block appliance resulted in a significant improvement in four of the five domains of the OSA-18 questionnaire, with a positive impact on the quality of life in growing children with OSA. Patients showed significant improvements in respiratory sleep parameters and parent-reported symptoms.

## Consent for publication

All undersigned authors, give their consent for the publication of the manuscript entitled (Effects of twin-block appliance on quality of life in OSA children with class 2 malocclusion and mandibular retrognathia) to be submitted in the Progress in Orthodontics.

## Ethical approval and consent to participate

This study was approved by the Research and Ethics Committee of the Health Campus at Universiti Sains Malaysia (approval number: Malaysia SM/JEPeM/20060315).

## Authors' contributions

Maen Zreaqat and Rozita Hassan designed and planned the study, Maen Zreaqat and Sahal Alforaidi collected the data, performed the measurements, and prepared the manuscript. All the authors commented, revised, and approved the final manuscript.

## Data availability

The data that support the findings of this study are available from the corresponding author upon reasonable request.

## Funding

This work was supported by Health Campus in Universiti Sains Malaysia under the RUI grant scheme 1001/PPSG/812158.

## Declaration of competing interest

The authors declare that they have no known competing financial interests or personal relationships that could have appeared to influence the work reported in this paper.
